# Malicious URL Detection Based on Associative Classification

**DOI:** 10.3390/e23020182

**Published:** 2021-01-31

**Authors:** Sandra Kumi, ChaeHo Lim, Sang-Gon Lee

**Affiliations:** 1 Department of Information Security, Dongseo University, Busan 47011, Korea; kumisandra54@gmail.com; 2 BITSCAN Co., Ltd., Seoul 04789, Korea; skscogh@naver.com

**Keywords:** data mining, web security, machine learning, malicious URLs, associative classification

## Abstract

Cybercriminals use malicious URLs as distribution channels to propagate malware over the web. Attackers exploit vulnerabilities in browsers to install malware to have access to the victim’s computer remotely. The purpose of most malware is to gain access to a network, ex-filtrate sensitive information, and secretly monitor targeted computer systems. In this paper, a data mining approach known as classification based on association (CBA) to detect malicious URLs using URL and webpage content features is presented. The CBA algorithm uses a training dataset of URLs as historical data to discover association rules to build an accurate classifier. The experimental results show that CBA gives comparable performance against benchmark classification algorithms, achieving 95.8% accuracy with low false positive and negative rates.

## 1. Introduction

The advancement of the World Wide Web (WWW) has attracted the attention of cybercriminals to use the web as a medium for distributing malware to compromise individuals’ and organizations’ networks. Attackers embed scripts, exploits, and executable files in online stores to steal user’s credit card data from websites.

According to the 2019 Kaspersky security bulletin [1], 85% of detected web threats were malicious universal resource locators (URLs). Malicious webpages attempt to install malware, collect sensitive information, and gain full access to victims’ devices. Drive-by downloads and social engineering are the most popular attacks that activate malicious URLs to disseminate malware. In a drive-by download attack, the attacker crafts malicious client-side scripting code (typically in JavaScript) to target a vulnerability in a web browser or plugin [2]. Cybercriminals have invented sophisticated ways such as advertising and breaking news to lure users into clicking on malicious links and open suspicious attachments [3]. When the user browses to the compromised site, the malicious script is executed, exploits a vulnerability in the web browser, and proceeds to download the malicious payload that gives attackers remote access to the victim’s computer. JavaScript-based attacks have been reported to account for a large fraction of web attacks in recent years [4].

Researchers have proposed defense approaches such as static analysis, dynamic analysis, blacklisting-based, and heuristic-based approaches for defending against malicious webpages [5]. Static analysis techniques [6,7] use statistical features to inspect websites without rendering the page in a browser. Dynamic analysis approaches such as Cuckoo [8] and SpyProxy [9] use a behavior analysis environment to detect malicious scripts. Attackers can easily identify the analysis environment, which increases their chances of evading the behavioral monitoring process. In blacklist-based methods, requested URLs are checked with predefined malicious URLs but not proactive in detecting newly emerging malicious webpages. Heuristic-based techniques create signatures of known attack payloads to scan websites. Unfortunately, systems based on predefined signatures are easily evaded by attackers and fail to detect new attacks [5].

Researchers have proposed many different data mining techniques to detect malicious URLs, but there are a limited number of works relating to the associative classification (AC) data mining approach. AC integrates two fields of data mining (classification and association rule mining) to build accurate and interpretable classifiers using association rules within a dataset. Classification rule mining discovers a small set of rules to form an accurate classifier [10]. Association rule mining aims at describing a dataset utilizing reliable association among patterns [11]. The rules produced in AC are easily interpreted by the end-user, unlike neural networks and probabilistic approaches, which produce classification models that are hard to understand [12]. AC also has the advantage of discovering useful hidden information within a dataset that can be missed by other classification models [13]. 

The few existing AC systems to detect malicious URLs focus on a specific attack, i.e., phishing. Abdelhamid et al. [14] and Jeeva and Rajsingh [15] proposed AC methods to detect phishing websites based on URL features. However, attackers can easily evade the analysis and detection of these systems by imitating the lexical features of benign URLs. When a malicious client-side code (e.g., JavaScript) is injected into a website, its URL is not affected. Thus, systems based on only URL features have a higher rate of false negatives. Hadi et al. [16] improved on the work of [14,15] to include content-based features but lacks the analysis of obfuscated JavaScript functions. Hence, a resilient and effective method to protect against malicious webpages is needed to keep pace with the evolution of malicious webpages. Our approach overcomes previous limitations by extracting features that effectively detect malicious URLs comprising phishing, malware, and drive-by-download websites.

In this paper, we use the classification based on association (CBA) algorithm to detect malicious URLs by utilizing both URL and webpage content features. CBA consists of two parts, a rule generator (CBA-RG) and a classifier builder (CBA-CB). CBA rule mining uses an a priori [11] algorithm to find the correlation between features extracted from webpages and builds a classifier based on rules that integrate classification with association rule mining. This knowledge discovery will help experts understand how malicious URLs evolve and the relationship between their attributes (features). Experimental results show that CBA rule mining achieved excellent performance on a real-world dataset, in terms of precision, recall, and false positive rate (FPR). 

This paper is organized as follows: Section 2 discusses related work. Section 3 gives the technical details of the proposed approach. We discuss the dataset used, experimental results, and performance comparison with existing approaches in Section 4. We outline our conclusions in Section 5.

## 2. Related Work

This section gives a brief description of related work in the detection of malicious webpages. In the past few years, research efforts have been made on the detection of malicious URLs using data mining approaches.

Eshete et al. presented a lightweight approach, called BINSPECT [5] that combines static analysis and emulation to apply supervised learning techniques in detecting malicious webpages. The experimental evaluation of BINSPECT achieved above 97% accuracy with low false signals.

Ma et al. [6] explored statistical methods from machine learning classifiers to detect malicious URLs based on lexical and host-based features of URLs. According to their experimental results, classifiers obtained 95-99% accuracy. Although this work achieves high detection accuracy, the extraction of host-based features is time-consuming which can cause a delay in real-time systems.

Curtsinger et al. proposed ZOZZLE [7], a low-overhead solution for detecting and preventing JavaScript malware. ZOZZLE uses a Bayesian classification of a hierarchical feature of the JavaScript abstract syntax tree to predict malware. According to their experimental evaluation on 1.2 million benign JavaScript samples, ZOZZLE achieved a low false-positive rate of 0.0003%.

Abdelhamid et al. [14] proposed multi-label classifier based associative classification (MCAC) to detect phishing websites. MCAC generates single and multi-label rules from a phishing training dataset to classify websites into legitimate or phishy.

Jeeva and Rajsingh [15] proposed an intelligent phishing URL detection model based on association rule mining. Fourteen URL features were exposed to associative rule mining apriori algorithm and predictive a priori algorithm. A URL is identified by using association rules in which the features are extracted to acquire unknown knowledge. Strong rules generated by the apriori algorithm with 100% confidence and by the predictive apriori algorithm with an accuracy level above 99% were considered for further analysis. Features such as transport layer security (TLS), unavailability of the top-level domain in the URL, and keyword within the path portion of the URL were frequent in phishing URLs. The experimental results show that the apriori algorithm mines rule faster than the predictive apriori algorithm. 93% of the phishing URLs are detected using the rules obtained by the apriori algorithm.

Kim et al. [17] proposed WebMon, machine learning, and YARA-based malicious webpage detection models. WebMon detects hidden exploit codes by tracing linked URLs to confirm whether the relevant websites are malicious. In this work, the authors focus on the features of Exploit Kits (EKs) and introduced 11 feature classes for the detection of malicious webpages. They tested six supervised learning classification algorithms (random forest, naive Bayes, logistic regression, Bayes net, J48, and SVM). Random forest achieved the best results; hence WebMon is built with the random forest learning algorithm. According to their experimental evaluation, WebMon has an accurate detection rate of 98% and 7.6 times faster than conventional malicious webpage detection tools.

Li et al. [18] presented a stacking model by combining gradient boosting decision tree, XGBoost, and LightGBM in multiple layers to detect phishing webpages using URL and HTML features. They evaluated their model on 50,000 webpages and achieved an accuracy of 97.30%, 4.46% on missing rate, and 1.61% on false alarm rate.

Blacklist approaches such as Google Safe Browsing [19] use a list of predefined malicious URLs list to detect malicious URLs. Cao et al. [20] proposed an automated individual white-list (AIWL), an anti-phishing tool based on legitimate login user interfaces (LUIs) of websites. AIWL uses the naive Bayes classifier to maintain a white-list of trusted websites visited by users and alerts whenever there is a possible attack. The drawback of the blacklist-whitelist based approach is that it cannot proactively detect new malicious webpages. 

## 3. Proposed Methodology

**Figure 1:**
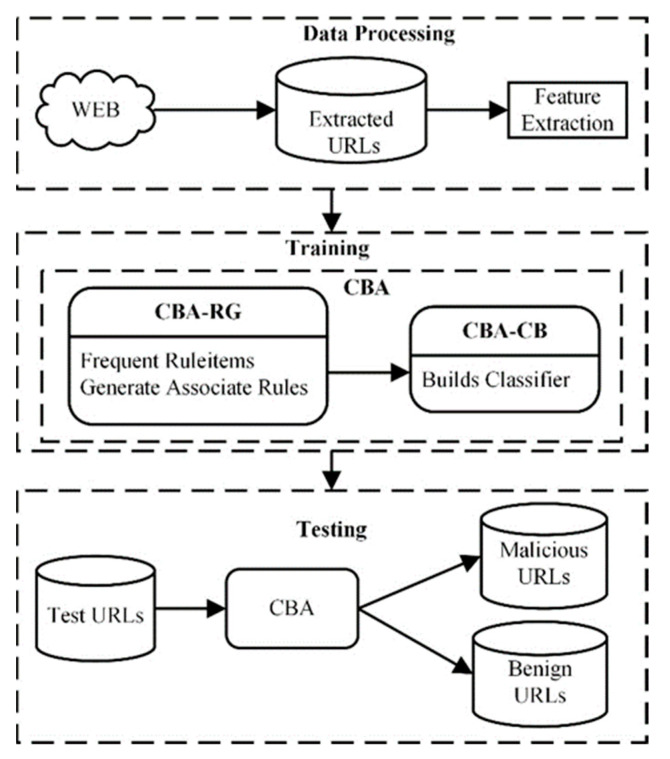
The architecture of the proposed approach.

Our proposed approach aims to analyze and classify a URL as malicious or benign. The architecture of our approach is shown in Figure 1. Firstly, we extract URL and content-based (HTML and JavaScript) features from URLs and then use the CBA model to train and make predictions. Extracting the features of the webpage helps in the detection of any malicious activity. Our approach considers malicious URLs related to phishing, malware, and drive-by-download websites. The following modules of our system are discussed below. 

### 3.1. Feature Extraction

Features are extracted from crawled URLs to classify them as malicious or benign. We extract two types of features from a URL: URL features and webpage’s content (HTML and JavaScript) features. The URL features are collected through the lexical scanning of the URL string. Webpage’s content features are extracted by visiting the webpage through Selenium WebDriver and headless Chrome Browser. Features extracted are described in the following subsections.

#### 3.1.1. URL Features

URL-based features can be divided into two categories; lexical and host-based features. We extract the lexical features (textual properties) URLs to distinguish the differences between malicious and benign URLs. We do not include host-based features such as IP address properties, WHOIS properties, and geographic properties because the extraction of these features is time-consuming. The URL features are introduced as follows:

(1) *Special Characters:* Attackers use special characters for URL encoded attacks to bypass validation logic. We count the number of special characters (‘;’, ‘+=’, ‘_’, ‘?’, ‘=’, ‘&’, ‘[‘, ‘]’, ‘#’, ‘~’, ‘%’, ‘@’, ‘$’, ‘*’, ‘+’, ‘!’, ‘|’) found in a URL.
(2) *Entropy of domain name:* Entropy measures the randomness factor or uncertainty in URLs; the higher the entropy, the higher the randomness factor in the URL. Entropy is used to detect randomized domain names. Some malicious URLs use domain generation algorithms (DGA) to change domains frequently, hence blacklisting these URLs is not efficient. DGA is a program that provides malware with new domains on-demand or on the fly [21]. URLs with high entropy are significant indicators of malicious behavior. Entropy can help detect malicious URLs by setting thresholds based on the entropies of legitimate URLs. We calculate the entropy of domain names, using the Shannon entropy formula:
  (1) $$H\left(x\right)\;=-\overset{n}{\underset{i=0}{\sum }}p(x_{i})\log_{b}p(x_{i})$$
  where *H*(*x*) is the Shannon entropy of string *x*, *b* is the base of the logarithm used, and *p*(*x*) is the probability mass function.
(3) *Sensitive words*: Sensitive words are mostly used in phishing URLs. We tokenize a URL to count the number of sensitive words in it, i.e., (’confirm’, ’account’, ’secure’, ’webscr’, ’banking’, ’login’, ‘signin’). Currently, our work only handles sensitive words in English Language.

#### 3.1.2. Webpage Content Features

HTML tags make it possible to disseminate malware by redirects users to compromised websites. Malicious JavaScript code consists of suspicious patterns and functions to launch drive-by-downloads and malware distribution. Attackers usually use JavaScript functions to evade detection. Redirect codes are injected into obfuscated JavaScript to hide redirect destinations. Attackers insert hidden links into web pages to trace the activities of users. The “location” object features are used to redirect users to malicious webpages. JavaScript functions commonly used by attackers include; *eval()*, *escape()*, *unescape()*, *replace()*, *exec()*, *ubound()*, etc. We use a combination of features proposed by the studies of Kim et al. [17] and Canali et al. [2]. Webpage features extracted in this work are briefly described below:

(1) *Webpage size-based features:* We extract two features under webpage size-based features; line counts and maximum line length. Malicious webpages are likely to have a long single line of code. In our dataset, we identified that 25% of malicious URLs with unique domain names had the same total number of lines of code. Benign webpages with a single line of code have a maximum line length of 6-469 characters. Hence, if the total number of line counts of code is 1 and the maximum line length is greater than 500 characters, the webpage is considered highly malicious.
(2) *Iframes:* Attackers use iframe tags to inject and load malicious code into webpages.
(3) *Elements with small area:* Attackers use high or low values for the height and width of tags to make attacks invisible to users.
(4) *JavaScript tags:* Webpage content is analyzed statistically for the presence of internal and external script tags on a webpage. Hackers embed malicious JavaScript code into websites to access sensitive user information. Injection of malicious JavaScript code can lead to CSRF vulnerability that allows the attacker to exploit the user’s browser cookies and permissions to perform malicious actions on a separate website. Benign websites mostly include external JavaScript for advertisement and analytics purposes. A malicious website with external JavaScript has the intention of redirecting users to compromised websites.
(5) *DOM functions:* Attackers use JavaScript to manipulate elements in a webpage’s DOM tree. DOM-modifying functions are used by hackers to tamper with data provided by users. We analyze source content to check for the presence of DOM functions such as appendChild, createElement, getElementByTagName and getElementById in a webpage.
(6) *Obfuscation and suspicious functions:* Attackers use obfuscation functions to evade detection, making code analysis difficult. They also include malicious JavaScript attachment, which runs through the Windows program, e.g., WScript.Shell. Suspicious functions such as ActiveXObject, CreateObject, CreateTextFile, FileSystemObject, and FileExists to create or access files and folders or create a backdoor to monitor activities on a computer.
(7) *Frequency of var and functions in JavaScript:* We check the frequency of var and function used in the JavaScript statement of webpages. var and function keywords occurrence is lower in malicious URLs since JavaScript code with these keywords is obfuscated or has a link attribute.
(8) *Whitespace ratio:* Whitespace obfuscation is the simplest obfuscation technique. Attackers include random whitespace inside source code to confuse static or automated analysis tools.

### 3.2. Detection of Malicious URLs using Classification Based on Association (CBA)

CBA [22] is an integrated algorithm that focuses on mining association rules in a given database called Class Association Rules (CARs) and builds a classifier based on the set of discovered CARs. The CBA algorithm consists of two phases:

(1) The Rule generator (CBA-RG) is based on the a priori algorithm to find all CARs that meet user-defined minimum support *(minsup)* and minimum confidence *(minconf)* thresholds. Support measures how frequent an itemset is in all transactions. Confidence is defined as the measure of certainty associated with each discovered itemset. Support and confidence can be calculated using the equations below. The a priori algorithm proposed by Agrawal et al. [11] is for mining frequent itemsets for Boolean association rules. In this work, CBA-RG iterates over historical extracted features of URLs (training dataset) to discover all frequent ruleitems, from which CARs are generated:
  (2) $$Support\left(A\right)=\frac{support\_count\left(A\right)}{total\;number\;of\;transactions}$$
  (3) $$Confidence\left(A\to B\right)=\frac{support\_count\left(A\cup B\right)}{support\_count\left(A\right)}$$
  Equation (2) expresses the support of ruleitems *A* as the ratio between the number of transactions containing *A* by the total number of transactions. Equation (3) expresses the confidence of a rule, where *A* is the antecedent, *B* is the consequence, support_count(A∪B) is the number of transactions containing the ruleitems A∪B, and support_count(A) is the number of transactions containing the rule items *A*.
  A rule item is of the form:〈condset, y〉, represents a rule: condset⇒y, where condset is a set of items, y∈Y is a class label (malicious or benign). For example, the following is a ruleitem to classify a URL:
  (4) {(A=1),(B=1)}⇒{class=benign}         
  where *A* and *B* are attributes (features). Rules are interpreted as “if-then” statements. In the example above, if A is 1 and B is 1, then the URL is classified as benign.
(2) The classifier builder (CBA-CB) builds a classifier using generated CARs. To build an accurate classifier, CBA-CB uses rule sorting and data coverage pruning procedure to discard redundant rules. Rules are selected to build a classifier based on ranking order, i.e., rules with high confidence and support are selected first. M1, a direct version of the CBA algorithm, and M2 are the pruning procedures used. M1 algorithm traverses the database multiple times to find the optimum number of rules to build an accurate classifier. The authors of CBA presented an improved version of the M1 algorithm (called M2) to reduce data access when the data is too large to be stored in the main memory. The available main memory and performance of computers have increased since the original paper was published, making the M2 algorithm less relevant [23]. Performance analysis by Jiří and Kliegr [24] showed that the M1 version of CBA is faster than M2 in most benchmarked combinations. In this study, we select the M1 algorithm as the pruning method to discard redundant rules. M1 algorithm, as shown in Algorithm 1, has three steps:
  **Step** **1:** Sort the generated rules *R* according to precedence operator (>).
  **Step** **2:** Select rules for the classifier according to the sorted sequence. The rules are iterated over the database, *D* to find instances that satisfy the rules. If a rule correctly classifies an instance in the database, it is marked and inserted at the end of the classifier, *C*. Instances that are correctly classified by selected rules are then removed from the database. A default class is selected, i.e., the majority class in the remaining instances to ensure that an instance is classified even if any other rule does not match it in the classifier.
  **Step** **3:** Discard rules in *C* that do not improve the accuracy of the classifier. These rules are discarded because they generated more errors.

A description of the CBA model to classify URLs is shown in Algorithm 2.

**Algorithm 1.** Pseudocode of M1 algorithm [22].Let R be the set of generated rules (CARs), and D is the training data. **Input**: Set of generated rules, R, Training data, D **Output**: Classifier, C
(1)R = sort(R)(2)for each rule r∈R in sequence do(3) temp= ∅;(4) for each instance d ∈D do(5)  if d satisfies the conditions of r then(6)   store d.id in temp and mark r if it correctly classifies d;(7) if r is marked then(8)   insert r at the end of C;(9)   delete all the instances with ids in temp from D;(10)   selecting a default class for the current C;(11)   compute the total number of errors of C;(12) end(13)end(14)Find the first rule p in C with the lowest total number of errors and drop all rules after p in C;(15)Add the default class associated with p to end of C(16)return C


**Algorithm 2.** Malicious URLs detection using CBA model.**Input**: Training data D, minsup, and minconf thresholds **Preprocess Data**: Discretize continuous features if any **Output**: Classification Results (Benign and Malicious URLs)Step 1: Rule Generator
**a.** Scan training data to discover frequent ruleitems**b.** Generate CARs R from frequent ruleitems, where $r_{i}\in R$
Step 2: Build Classifier
**a.** Sort rule set according to confidence and support**b.** Cr: Scan D to find instances covered by $r_{i}$ and correctly classifies an instance in D**c.** Build a classifier C using rules in Cr
Step 3: Classification of URLs**a.** Use C to classify test data.


## 4. Experimental Setup and Evaluation

### 4.1. Evaluation Metrics

We use precision, recall, confusion matrix, and accuracy as performance metrics to evaluate the performance of our proposed approach:

(1) *Confusion Matrix (C).* It is used to evaluate the accuracy of a classifier. The confusion matrix contains predicted and actual classifications done by a classifier.
  (5) $$C=\left[\begin{array}{cc} TN & FP\\ FN & TP \end{array}\right]\;\;\;\;\;$$
  - True Positives (TP): correct malicious URLs prediction.
  - True Negatives (TN): correct benign URLs prediction.
  - False Positives (FP): incorrect malicious URLs prediction.
  - False Negatives (FN): incorrect benign URLs prediction.
(2) *Accuracy*. This is the ratio of correct predictions to the total number of samples:
  (6) $$Accuracy=\frac{TP+TN}{TP+TN+FP+FN}$$
(3) *Precision.* This is defined as the number of true positives over the number of true positives plus the number of false positives:
  (7) $$Precision=\frac{TP}{TP+FP}$$
(4) *Recall.* This is defined as the number of true positives over the number of true positives plus the number of false negatives.
  (8) $$Recall=\frac{TP}{TP+FN}$$

### 4.2. Evaluation of Proposed Approach

We used an Intel ® Core ™ i5-7500 CPU @ 3.40 GHz desktop and 64-bit Windows 10 operating system with 16 GB of memory for all experiments in this paper. A dataset of benign and malicious URLs was collected from benchmark sources. Benign URLs were collected by crawling Alexa’s top 500 sites [25]. Malicious URLs were collected from OpenPhish [26], VxVault [27] and URLhaus [28]. We labeled collected benign and malicious URLs as B and M, respectively. We utilized Python built-in libraries to parse and extract features from URLs. We transformed feature extraction results into a .csv file. The CBA model to classify URLs was implemented in R using the arulesCBA R package [29]. The CBA algorithm was evaluated using 10-fold cross-validation to test 1200 labeled URLs, made up of 700 malicious and 500 benign URLs. The CBA algorithm uses our dataset as a historical database of URLs to discover rules associated with benign and malicious URLs. Minimum support *(minsup)* has a strong effect on the accuracy of the classifier; if *minsup* is set too high, CARs may fail to cover all instances in the training dataset. The following grid values of support and confidence threshold were investigated to select the best threshold for generating CARs: Gridsupport = [1, 2, 3, 4, 5] and Gridconfidence = [50, 60, 70, 80, 90, 100]. Table 1 shows the accuracy of different support and confidence thresholds. We observed that if *minsup* is lowered to 1–2%, with 90% confidence, the classifier has better performance. In our work, *minsup* is set to 1% and 90% for minimum confidence *(minconf)* and maximum rule length of 3 to discover CARs. By applying these settings of parameters on our dataset, 52 rules were mined to analyze URLs, with the default class to be malicious. 

Continuous (numerical) values of extracted features are discretized before generating CARs. Because AC works with association rules, and data needs to be converted into binary or categorical items. Discretization is a data transformation where continuous-valued attributes (features) are converted into categorical data. CBA algorithm discretizes continuous-valued features using the Entropy method proposed by Fayyad and Irani [30]. Continuous-valued attributes (features) are discretized by partitioning the range of features into subranges. The discretization of continuous-valued attributes will not be discussed in this paper (see Fayyad and Irani [30] for more details). Table A1 of Appendix A shows an extract of mined CARs to classify URLs. It also gives the Support and Confidence of the CARs. Support is how frequent ruleitems appear in the training data, and confidence represents the certainty of CARs generated. From our experiment results, the CBA model achieved an accuracy rate of 95.83% with low false positive and negative rates in the classification of URLs.

Table 2 shows the impact analysis of features in the classification of URLs. We observed that entropy of domain name, JavaScript tags, DOM functions, whitespace percentage, and the frequency of var and function were the top five important features.

### 4.3. Performance Comparison with Existing Works

We compare our work with the authors of [15], which uses association rules to detect malicious URLs. Unlike our approach, they extracted only lexical features from URLs and focused on a specific type of malicious URLs. We performed the experiments of [15] in WEKA using the parameters specified by the authors on our dataset. The comparison results are shown in Table 3. Although their approach shows good performance, it lacks the analysis and detection of obfuscated JavaScript code, which is the primary form of attack used in drive-by downloads. As shown in Table 3, the work of [15] has a high false negative rate of 7.57% compared to our approach in the classification of URLs, making it less effective when used alone to detect malicious webpages. Attackers can imitate the textual features of benign URLs, thus, URL features should be combined with content-based features (HTML and JavaScript) to increase the performance of the system. 

We also compared well-known classification algorithms (support vector machine (SVM), naive Bayes, and logistic regression) to evaluate the performance of CBA. These classifiers were used in the work of J. Ma et al. [6] to detect malicious websites. SVM constructs a linear hyperplane that separates the dataset into different classes. SVM finds the optimal solution by maximizing the hyperplane’s margin. *Libsvm* library was used in this work. Naive Bayes is a probabilistic classifier based on Bayes’ theorem with an assumption of independence among predictors. Logistic regression is an extension of linear regression. It is a statistical method for predicting binary classification, where the dependent variable is categorical. In this study, we use the binary logistic regression, where the target variable has two outcomes that are benign or malicious URL. The experiments of SVM, naive Bayes, and Logistic regression were performed in WEKA [31]. We maintained the default setting of all the classifiers provided in WEKA API for our dataset. We compare the precision, recall, ROC area, and FPR of the classifiers mentioned above with CBA on our dataset, using 10-fold cross-validation. As shown in Table 4, naïve Bayes performed efficiently, but CBA outperformed it with a precision of 91.30 and 97.67% recall. Logistic regression and SVM have high FPR, i.e., 19.2% and 16.2%, respectively. 

Overall CBA yields good performance over benchmark classifiers as shown in Table 4. It also gives the interpretability of results which will help security analysts and end-users to understand how malicious URLs evolve. 

## 5. Conclusions

Cybercriminals have invented sophisticated ways such as injecting malicious code into websites to disseminate malware in an attempt to infect target systems. Associative classification approaches to detect malicious URLs mainly focus on phishing websites. Regarding this, we present an approach based on classification based on association (CBA) algorithm to detect malicious URLs comprising phishing, malware, and drive-by-download websites. We have extracted URL and webpage content-based features that effectively identify benign and malicious URLs. Our experimental analysis shows that the CBA algorithm achieved comparable performance on a real-world dataset against benchmark classification models. 

## Figures and Tables

**Table 1:** Accuracy of 10-fold cross-validation of the proposed approach over different values of minsup and minconf thresholds.

Support (*minsup*) %	Confidence (*minconf*) %					
	50	60	70	80	90	100
1	91.67	91.67	91.67	91.67	**95.8**	83.33
2	92.5	92.5	92.5	92.5	92.5	68.33
3	90	90	90	90	90	60
4	90	90	90	90	90	60
5	86.67	85	84.17	88.33	90	60

**Table 2:** Importance of features.

Feature	Score
Entropy of domain name	0.419320
JavaScript Tags	0.190276
DOM functions	0.075120
Whitespace percentage	0.073060
Frequency of var, function keyword	0.065900
Special character ratio	0.042401
Line counts	0.040867
Iframe counts	0.040005
Maximum line length	0.024783
Obfuscation and suspicious functions	0.019935
Elements with small area	0.007263
Sensitive words	0.001088

**Table 3:** Comparison with existing work.

Approach	Accuracy	Precision	Recall	False Negative Rate
Jeeva et al. [15]	95.8	96.2	95.8	7.57
**Our Method**	**95.8**	**91.3**	**97.67**	**1.35**

**Table 4:** Comparison of CBA (Our Approach) with other classification models.

Classifier	Precision (%)	Recall (%)	ROC Area (%)	False Positive Rate (%)
SVM	83.4	80	81.9	16.2
Naive Bayes	90.9	90.3	95.2	8.4
Logistic	85.9	84.8	94.1	19.7
Our Approach (CBA)	**91.30**	**97.67**	**96.2**	**8.6**

## Appendix A

**Table A1:** An extract of CARs generated by the CBA algorithm to classify URLs.

CARs	Support (%)	Confidence (%)
**{Entropy = [3.78, inf]}** ⇒ **{Label = M}**	48.51	100
**{special characters = [0.00336, 0.0619]}** ⇒ **{Label = M}**	24.62	100
**{var_function frequency = [59.5,904), DOM functions = [12.5,148)}** ⇒ **{Label =B}**	13.79	100
**{line counts = [570,688), maximum line length = [888, 1.16e+03)}** ⇒ **{Label =M}**	12.22	100
**{whitespace %= [1.0258,1.1005), DOM functions = [-Inf,1.5)}** ⇒ **{Label = M}**	11.85	100
**{JavaScript tags= [2.5,27.5), Entropy= [-Inf,3.03)}** ⇒ **{Label =B}**	11.48	100
{DOM functions = [12.5,148), Entropy= [-Inf,3.03)} ⇒ {Label =B}	9.25	100
{whitespace %= [1.0258,1.1005), Entropy = [3.03,3.66)} ⇒ {Label = B}	9.07	100
{line counts = [11.5,570), Entropy = [-Inf,3.03)} ⇒ {Label =B}	6.75	100
{Elements with small area = [0.5,Inf), var_function frequency = [59.5,904) } ⇒ {Label =B}	5.46	100
{iframe counts = [3.5,Inf), Entropy = [3.03,3.66)} ⇒ {Label =B}	5.73	100
{line counts = [11.5,570), var_function frequency = [59.5,904) } ⇒ {Label =B}	4.81	100
{maximum line length = [4.01e+03,4.09e+03]} ⇒ {Label =M}	3.88	100
{maximum line length = [1.16e+03, 4.01e+03), Entropy = [-Inf,3.03)} ⇒ {Label =B}	3.42	100
{Obfuscation and suspicious functions = [10.5,Inf),var_function frequency = [2.5,59.5)} ⇒ {Label =M}	2.50	100
{whitespace %= [0.99362,1.0047), DOM functions = [1.5,7.5)} ⇒ {Label = B}	2.31	100
{Elements with small area = [-Inf,-0.5)} ⇒ {Label =M}	1.94	100
{iframe counts = [-Inf,0.5), Obfuscation and suspicious functions = [10.5,Inf),} ⇒ {Label =M}	1.75	100
{maximum line length = [-Inf,141), JavaScript tags = [2.5,4.5)} ⇒ {Label =B}	1.67	100
{whitespace %= [1.1487,Inf), sensitive word = [-Inf,0.5)} ⇒ {Label =B}	1.48	100
{whitespace %= [-Inf,0.17415), special characters = [-Inf,0.00336)} ⇒ {Label =M}	1.48	100
{iframe counts = [-Inf,0.5), whitespace % = [1.0322,1.1487)} ⇒ {Label =M}	12.12	99.24
{iframe counts = [3.5,Inf),special characters = [-Inf,0.00336)} ⇒ {Label =B}	9.90	99.07
{whitespace % = [1.0322,1.1487), special characters = [-Inf,0.00336]} ⇒ {Label =M}	8.51	98.92
{whitespace % = [0.99362,1.0047), Entropy = [-Inf,3.03)} ⇒ {Label =B}	6.94	98.62
{DOM functions = [12.5,148),special characters = [-Inf,0.00336)} ⇒ {Label =B}	18.61	98.52
{maximum line length = [5.27e+03,1.83e+04), JavaScript tags = [-Inf,0.5)} ⇒ {Label =M}	3.79	97.61
